# Causal relationship between gut microbiome, immune cell, and systemic lupus erythematosus: A Mendelian randomization analysis

**DOI:** 10.1097/MD.0000000000043703

**Published:** 2025-08-01

**Authors:** Hongye Wang, Jia Zhang, Mifeng Yang, Jiyu Chen, Xiran Yang, Na Yang, Bo Zhao

**Affiliations:** a Clinical Medical College of Dali University, Dali, Yunnan Province, China; b Nephrology, Rheumatology and Immunology Department, Kunming Children’s Hospital, Kunming, Yunnan Province, China.

**Keywords:** casual effect, gut microbiome, immune cell, Mendelian randomization analysis, systemic lupus erythematosus

## Abstract

Systemic lupus erythematosus (SLE) is a highly heterogeneous, multifactorial complex disease. Its pathogenic mechanisms are extremely intricate, primarily characterized by immune dysregulation caused by the combined influence of genetic, environmental, and biological factors. In recent years, increasing evidence has shown that gut microbiome play a crucial role in the onset and progression of SLE. However, no study has yet specifically elucidated the mechanisms by which gut microbiome influence SLE. Based on the central role of immune cells in immune balance and the pathogenesis of SLE, we propose the hypothesis that gut microbiome affect the onset and progression of SLE through the regulation of immune cells. To test this hypothesis, we utilized large-scale genome-wide association studies data from a cohort of gut microbiome, 713 immune cell types, and SLE patients. We conducted large-scale Mendelian randomization and mediation analyses to explore the causal relationships between gut microbiota, immune cells, and SLE. The results indicate that immune cells mediate the causal relationship between gut microbiome and SLE, and 2 specific upstream and downstream regulatory mechanisms were identified. These findings provide new insights and a theoretical foundation for the development of therapeutic targets for SLE.

## 1. Introduction

Systemic lupus erythematosus is a complex, multisystem autoimmune disease with intricate pathogenesis, involving multiple factors such as genetic and epigenetic influences.^[1]^ In recent years, the relationship between gut microbiome and systemic lupus erythematosus (SLE) has garnered significant attention. Studies have shown that the gut microbiome plays a critical role in the pathogenesis of SLE.^[2]^ The composition of the gut microbiome in SLE patients undergoes significant alterations, with a notable decrease in microbial diversity compared to healthy individuals. Changes in specific microbiota are associated with disease activity and elevated levels of anti-double-stranded DNA antibodies in patients.^[3,4]^ Consequently, targeting specific gut microbiome represents a promising therapeutic approach for the treatment of SLE.^[5]^ However, the precise mechanisms by which the gut microbiome influences the onset and progression of SLE, particularly in relation to immune balance, remain unclear, hindering the development of effective therapeutic strategies.

As important carriers of immune balance, immune cells may serve as critical mediators through which the gut microbiome influences immune balance, thereby affecting the development of SLE. Immune cells play a central role in the pathogenic mechanisms of SLE, acting as common carriers for the regulation of immune balance by various pathogenic factors.^[6]^ Studies have shown that the gut microbiome can influence immune balance either by triggering cross-immune reactions through molecular mimicry or by modulating the function of specific immune cells through its metabolic products.^[7,8]^ This suggests that immune cells may be important intermediaries through which the gut microbiome regulates immune balance, thereby participating in the onset and progression of autoimmune diseases, such as SLE. However, due to the diversity of both the gut microbiome and immune cells, there is currently no suitable approach to specifically investigate the relationship and mechanisms between particular microbiota and immune cells.

Mendelian randomization (MR) is an important method used in epidemiological research to assess causal effects. This approach utilizes single nucleotide polymorphisms (SNPs) as an instrumental variable (IV) to evaluate the causal relationships and effects between different exposures and outcomes.^[9]^ This method provides an excellent option for selecting suitable research targets and contributes to the gradual elucidation of the specific mechanisms through which the gut microbiome influences autoimmune diseases such as SLE. Based on this, we pioneered the use of a genome-wide association studies (GWAS) cohort of gut microbiome, 713 immune cell types, and SLE patients, conducting large-scale MR and mediation analyses. The aim was to investigate the causal relationships between the gut microbiome, immune cells, and SLE, and to explore whether specific gut microbiota affect the onset and development of SLE by regulating immune cells. Our results indicated that FUCCAT.PWY.fucose.degradation increased the risk of SLE by enhancing the expression of central memory CD4+ T cells (CD4 on CM CD4+), while Lachnospiraceae bacterium 3_1_46FAA reduced the risk of SLE by decreasing the expression of CD25 on CD39+ regulatory T (Treg) cells. As the first study to explore the relationship between the gut microbiome, immune cells, and SLE, our findings provide new insights for the development of therapeutic targets for SLE and lay a theoretical foundation for the development of related diagnostic and treatment methods.

## 2. Methods and materials

### 2.1. Research design and data sources

#### 2.1.1. Research design

**Figure 1.**
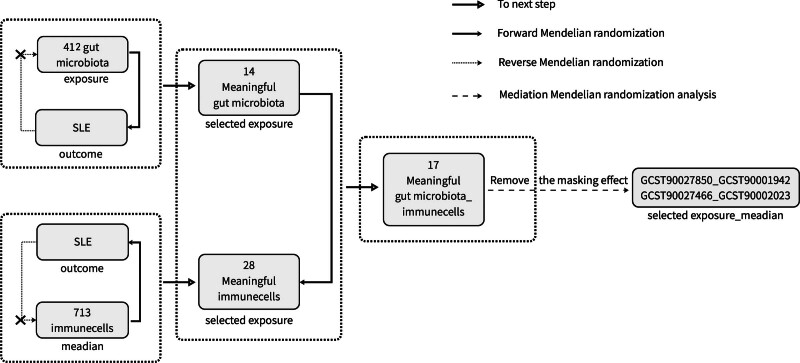
Research workflow diagram. SLE = systemic lupus erythematosus.

This study employs bidirectional MR (BMR) and mediation MR analysis methods to assess the causal effects of gut microbiome exposures on SLE outcomes. Additionally, it investigates whether 713 immune cell types serve as mediators in this causal pathway.The research flowchart is shown in Fig. 1.

#### 2.1.2. Data sources

The data for this study were obtained from publicly available summary statistics from GWAS. The SLE-GWAS data were sourced from a large-scale Finnish cohort, which inclu des 1306 SLE patients and 372,273 control samples, resulting in a total sample size of 373,579. This cohort provides genome-wide SNPs data related to SLE.^[10]^ The gut microbiome GWAS data come from a study by Esteban A. Lopera-Maya et al., which involved 7738 Dutch individuals and examined 207 microbial taxa and 205 bacterial metabolic pathways.^[11]^ The immune cells GWAS data were derived from a study by Valeria Orrù et al., which involved 3757 Sardinian individuals and investigated the genome-wide association of 731 immune cell expression levels.^[12]^

### 2.2. Selection and extraction of instrumental variables

IVs are critical for conducting MR analysis. We selected SNPs associated with 412 types of gut microbiome and 713 significant traits using a threshold of *P* < 1e−5. We then calculated the linkage disequilibrium for these SNPs (with a 10,000 kb window) and removed SNPs with an *r*² < 0.001 to exclude those with insufficient linkage disequilibrium. Finally, we discarded SNPs with *F*-statistics lower than 10 and palindromic SNPs. The remaining SNPs were used as IVs for further analysis.^[5]^

### 2.3. Mendelian randomization analysis

#### 2.3.1. Screening of gut microbiome exposures related to systemic lupus erythematosus outcomes and estimation of causal effects

The inverse variance weighted method (IVW) was used as the primary method for causal effect analysis, with gut microbiome as the exposure and SLE as the outcome. Causal effects of 412 types of gut microbiome on SLE were estimated. The weighted median method and MR-Egger regression were employed for pleiotropy analysis, and Cochran Q test was used for heterogeneity analysis to assess the robustness of the results. Bayesian algorithms were applied to validate the causal relationship between the selected gut microbiota and SLE. Additionally, reverse MR was conducted to exclude reverse causality and confounding effects.

#### 2.3.2. Screening of immune cell exposures related to systemic lupus erythematosus outcomes and estimation of causal effects

IVW was used as the primary method for causal effect analysis, with 713 types of immune cells as the exposure and SLE as the outcome. Causal effects of the 713 immune cells on SLE outcomes were estimated. The weighted median method and MR-Egger regression were employed for pleiotropy analysis, and Cochran Q test was used for heterogeneity analysis to assess the robustness of the results. Bayesian algorithms were applied to validate the causal relationship between the selected immune cells and systemic lupus erythematosus. Additionally, reverse MR was conducted to exclude reverse causality and confounding effects.

#### 2.3.3. Estimation of causal effects of gut microbiome exposure on immune cell outcomes

IVW was used as the primary method for causal effect analysis, with the selected gut microbiome as the exposure and the selected immune cells as the outcome. The causal effects of each selected gut microbiome type on each selected immune cell were estimated. Additionally, the weighted median method and MR-Egger regression were applied to perform sensitivity analyses for each association, in order to assess the robustness of the results.

### 2.4. Mediation effect analysis

#### 2.4.1. Calculation of mediation effect

The mediation effect is calculated using the product method, which involves multiplying the causal effect of the exposure on the mediator variable (β1) by the causal effect of the mediator variable on the outcome (β2), resulting in the product (β1 × β2).

#### 2.4.2. Proportion of mediation effect

The proportion of the mediation effect in the total causal effect is calculated using the formula (β1 × β2)/βa, where βa represents the total causal effect of the exposure on the outcome.

### 2.5. Sensitivity analysis

#### 2.5.1. Pleiotropy test

The presence of pleiotropy in the genetic IVs is tested using the MR-Egger regression and MR-PRESSO methods.

#### 2.5.2. Heterogeneity test

Cochran Q test is used to assess the heterogeneity of the IVW results.

#### 2.5.3. Leave-one-out analysis

Each SNP is sequentially excluded to evaluate the influence of each SNP in the exposure on the outcome.

### 2.6. Statistical software and tools

All analyses were performed using R software (version 4.4.2), primarily relying on the “TwoSampleMR,” “MendelianRandomization,” and “mediation” packages. The significance level was set at *P* < .05.

### 2.7. Ethical review

This study used publicly available summary-level GWAS data. No individual-level data were collected. Institutional review board approval was waived per international guidelines for de-identified genomic analyses.

## 3. Results

### 3.1. Causal effect between gut microbiome exposure and systemic lupus erythematosus outcomes

We first employed a BMR approach to identify gut microbiome types associated with causal effects on SLE outcomes. Using the IVW method, we identified 15 gut microbiome types from a pool of 412 that demonstrated significant causal effects on SLE (see Table S1, Supplemental Digital Content, https://links.lww.com/MD/P582). Heterogeneity and pleiotropy analyses revealed no evidence of pleiotropy or heterogeneity in these associations (*P* > .05). Subsequent Bayesian validation excluded one gut microbiota species with a *P*-value >.05 (see Table S2, Supplemental Digital Content, https://links.lww.com/MD/P582). Reverse MR analysis further confirmed that the remaining 14 gut microbiome types did not exhibit reverse causal effects on SLE (see Table S3, Supplemental Digital Content, https://links.lww.com/MD/P582). Consequently, we identified 14 gut microbiome types with a robust causal effect on SLE, as detailed in Table 1.

**Table 1:** Fifteen selected gut microbiome types associated with causal effects on systemic lupus erythematosus.

Exposure	Method	nsnp	beta	or	or_lci95	or_uci95	pval
GCST27466	MR Egger	10	‐0.19	0.83	0.27	2.53	.752
	Weighted median	10	0.40	1.49	0.97	2.29	.067
	**IVW**	**10**	**0.39**	**1.48**	**1.07**	**2.05**	**.017**
GCST27473	MR Egger	4	‐0.34	0.71	0.13	3.90	.732
	Weighted median	4	‐0.75	0.47	0.25	0.91	.026
	**IVW**	**4**	‐**0.64**	**0.53**	**0.31**	**0.88**	**.015**
GCST27558	MR Egger	15	‐0.62	0.54	0.24	1.22	.162
	Weighted median	15	‐0.17	0.84	0.65	1.09	.194
	**IVW**	**15**	‐**0.26**	**0.77**	**0.63**	**0.95**	**.015**
GCST27585	MR Egger	6	‐0.13	0.88	0.10	7.66	.913
	Weighted median	6	0.19	1.21	0.71	2.07	.474
	**IVW**	**6**	**0.45**	**1.57**	**1.04**	**2.36**	**.031**
GCST27601	MR Egger	6	‐0.12	0.88	0.27	2.90	.847
	Weighted median	6	0.36	1.44	0.93	2.21	.100
	**IVW**	**6**	**0.34**	**1.41**	**1.02**	**1.96**	**.040**
GCST27684	MR Egger	5	0.30	1.34	0.45	3.99	.631
	Weighted median	5	0.43	1.54	0.92	2.56	.099
	**IVW**	**5**	**0.49**	**1.63**	**1.07**	**2.49**	**.023**
GCST27745	MR Egger	5	0.30	1.34	0.45	3.99	.631
	Weighted median	5	0.43	1.54	0.88	2.67	.127
	**IVW**	**5**	**0.49**	**1.63**	**1.07**	**2.49**	**.023**
GCST27757	MR Egger	11	‐0.13	0.88	0.24	3.28	.856
	Weighted median	11	‐0.40	0.67	0.46	0.98	.041
	**IVW**	**11**	‐**0.40**	**0.67**	**0.50**	**0.90**	**.008**
GCST27790	MR Egger	13	0.13	1.13	0.34	3.77	.841
	Weighted median	13	‐0.33	0.72	0.50	1.03	.069
	**IVW**	**13**	‐**0.27**	**0.76**	**0.59**	**0.98**	**.037**
GCST27797	MR Egger	9	0.64	1.89	0.34	10.48	.488
	Weighted median	9	0.30	1.35	0.87	2.10	.182
	**IVW**	**9**	**0.38**	**1.47**	**1.04**	**2.08**	**.031**
GCST27828	MR Egger	17	0.14	1.15	0.58	2.28	.685
	Weighted median	17	‐0.30	0.74	0.60	0.92	.007
	**IVW**	**17**	‐**0.22**	**0.80**	**0.68**	**0.94**	**.006**
GCST27850	MR Egger	4	‐0.35	0.70	0.19	2.65	.655
	Weighted median	4	‐0.45	0.64	0.44	0.93	.020
	**IVW**	**4**	**‐0.40**	**0.67**	**0.50**	**0.91**	**.011**
GCST27853	MR Egger	17	‐0.15	0.86	0.55	1.35	.522
	Weighted median	17	‐0.19	0.83	0.71	0.97	.018
	**IVW**	**17**	‐**0.14**	**0.87**	**0.77**	**0.98**	**.024**
GCST27854	MR Egger	8	‐0.88	0.42	0.01	20.33	.674
	Weighted median	8	0.35	1.42	0.87	2.32	.156
	**IVW**	**8**	**0.43**	**1.53**	**1.06**	**2.22**	**.025**

The bold values highlight the results of the primary analysis method employed in our study, namely the inverse-variance weighted (IVW) method. This is the standard and often most reliable approach for the main analysis in Mendelian randomization studies.

beta = the estimated causal effect of the exposure factor on the outcome, Exposure = ID sequences of the gut microbiome type, IVW = inverse-variance weighted, nsnp = the number of instrumental variable SNPs, or = odds ratio, or_lci95 = the lower bound of the 95% confidence interval for the odds ratio, or_uci95 = the upper bound of the 95% confidence interval for the odds ratio, pval = *P*-value.

### 3.2. Causal effects of immune cells exposure on systemic lupus erythematosus

We next employed the same BMR approach to identify immune cells with a causal effect on SLE outcome. Using the IVW method, we identified 39 immune cells out of a total of 713 that exhibited a causal relationship with SLE, as detailed in Table S4, Supplemental Digital Content, https://links.lww.com/MD/P582. All these associations passed pleiotropy and heterogeneity tests. Following Bayesian validation, 10 immune cells with *P*-values > .05 were excluded, as indicated in Table S5, Supplemental Digital Content, https://links.lww.com/MD/P582. Reverse MR further revealed that one immune cell from the remaining results exhibited an inverse causal effect on SLE, as shown in Table S6, Supplemental Digital Content, https://links.lww.com/MD/P582. Consequently, after removing immune cells with inverse causal effects, we identified 28 immune cells that demonstrate a valid causal relationship with SLE, as presented in Table 2.

**Table 2:** Twenty-eight types immune cells associated with causal effects on systemic lupus erythematosus.

Exposure	Method	nsnp	beta	or	or_lci95	or_uci95	pval
**ebi-a-GCST90001513**	**MR Egger**	10	0.28	1.33	1.10	1.60	.017
	**Weighted median**	10	0.18	1.20	1.03	1.40	.021
	**IVW**	**10**	**0.14**	**1.15**	**1.01**	**1.30**	**.034**
**ebi-a-GCST90001596**	**MR Egger**	14	0.05	1.05	0.82	1.36	.687
	**Weighted median**	14	-0.10	0.90	0.72	1.14	.395
	**IVW**	**14**	**-0.16**	**0.85**	**0.74**	**0.99**	**.037**
ebi-a-GCST90001604	MR Egger	9	0.77	2.16	0.70	6.71	.223
	Weighted median	9	0.41	1.51	1.09	2.10	.013
	**IVW**	**9**	**0.41**	**1.50**	**1.11**	**2.03**	**.009**
ebi-a-GCST90001611	MR Egger	9	0.17	1.19	0.70	2.03	.548
	Weighted median	9	0.16	1.17	0.93	1.48	.187
	**IVW**	**9**	**0.19**	**1.21**	**1.01**	**1.46**	**.041**
ebi-a-GCST90001640	MR Egger	6	-1.10	0.33	0.07	1.50	.226
	Weighted median	6	-0.53	0.59	0.40	0.86	.006
	**IVW**	**6**	**-0.54**	**0.58**	**0.41**	**0.83**	**.003**
ebi-a-GCST90001666	MR Egger	33	-0.03	0.97	0.94	0.99	.019
	Weighted median	33	-0.05	0.95	0.92	0.99	.005
	**IVW**	**33**	**-0.04**	**0.96**	**0.94**	**0.99**	**.002**
ebi-a-GCST90001680	MR Egger	12	0.23	1.26	0.96	1.65	.122
	Weighted median	12	0.17	1.19	0.96	1.47	.123
	**IVW**	**12**	**0.17**	**1.19**	**1.02**	**1.38**	**.024**
ebi-a-GCST90001704	MR Egger	6	-0.07	0.93	0.77	1.14	.541
	Weighted median	6	-0.13	0.88	0.73	1.07	.199
	**IVW**	**6**	**-0.18**	**0.84**	**0.72**	**0.98**	**.029**
ebi-a-GCST90001705	MR Egger	8	-0.06	0.95	0.79	1.14	.581
	Weighted median	8	-0.10	0.90	0.75	1.08	.265
	**IVW**	**8**	**-0.15**	**0.86**	**0.74**	**1.00**	**.048**
ebi-a-GCST90001710	MR Egger	5	-0.15	0.86	0.22	3.32	.843
	Weighted median	5	-0.29	0.75	0.51	1.10	.136
	**IVW**	**5**	**-0.38**	**0.69**	**0.52**	**0.91**	**.009**
ebi-a-GCST90001711	MR Egger	4	-0.11	0.90	0.73	1.10	.419
	Weighted median	4	-0.25	0.78	0.64	0.95	.014
	**IVW**	**4**	**-0.25**	**0.78**	**0.63**	**0.96**	**.020**
ebi-a-GCST90001753	MR Egger	19	-0.23	0.79	0.58	1.09	.166
	Weighted median	19	-0.21	0.81	0.67	1.00	.046
	**IVW**	**19**	**-0.26**	**0.77**	**0.63**	**0.95**	**.013**
ebi-a-GCST90001758	MR Egger	17	-0.10	0.91	0.61	1.34	.631
	Weighted median	17	-0.21	0.81	0.64	1.04	.098
	**IVW**	**17**	**-0.19**	**0.83**	**0.70**	**0.98**	**.024**
ebi-a-GCST90001799	MR Egger	13	-0.03	0.97	0.90	1.04	.385
	Weighted median	13	-0.05	0.95	0.88	1.03	.196
	**IVW**	**13**	**-0.06**	**0.94**	**0.89**	**1.00**	**.038**

Exposure	Method	nsnp	b	or	or_lci95	or_uci95	pval
ebi-a-GCST90001841	MR Egger	17	0.09	1.09	0.94	1.26	.265
	Weighted median	17	0.03	1.03	0.92	1.16	.607
	**IVW**	**17**	**0.11**	**1.11**	**1.02**	**1.22**	**.018**
ebi-a-GCST90001847	MR Egger	16	0.00	1.00	0.89	1.13	.985
	Weighted median	16	0.04	1.04	0.91	1.18	.598
	**IVW**	**16**	**0.10**	**1.11**	**1.01**	**1.22**	**.034**
ebi-a-GCST90001870	MR Egger	9	-0.09	0.92	0.84	1.00	.087
	Weighted median	9	-0.08	0.92	0.85	1.00	.041
	**IVW**	**9**	-**0.09**	**0.92**	**0.86**	**0.98**	**.007**
ebi-a-GCST90001928	MR Egger	9	0.06	1.06	0.84	1.34	.646
	Weighted median	9	0.11	1.11	0.93	1.33	.238
	**IVW**	**9**	**0.16**	**1.18**	**1.03**	**1.35**	**.020**
ebi-a-GCST90001942	MR Egger	12	0.06	1.06	0.99	1.15	.144
	Weighted median	12	0.05	1.05	0.97	1.14	.238
	**IVW**	**12**	**0.06**	**1.07**	**1.00**	**1.14**	**.046**
ebi-a-GCST90001946	MR Egger	10	-0.10	0.91	0.77	1.07	.282
	Weighted median	10	-0.16	0.85	0.74	0.99	.036
	**IVW**	**10**	-**0.17**	**0.85**	**0.76**	**0.94**	**.001**
ebi-a-GCST90001995	MR Egger	17	-0.11	0.90	0.70	1.15	.418
	Weighted median	17	-0.18	0.83	0.72	0.97	.018
	**IVW**	**17**	-**0.12**	**0.89**	**0.80**	**0.99**	**.030**
ebi-a-GCST90001997	MR Egger	21	-0.13	0.88	0.74	1.04	.160
	Weighted median	21	-0.14	0.87	0.76	0.99	.039
	**IVW**	**21**	**-0.09**	**0.91**	**0.83**	**1.00**	**.039**
ebi-a-GCST90002019	MR Egger	14	-0.03	0.97	0.89	1.07	.557
	Weighted median	14	-0.05	0.95	0.87	1.04	.298
	**IVW**	**14**	**-0.09**	**0.91**	**0.85**	**0.97**	**.006**
ebi-a-GCST90002021	MR Egger	12	-0.07	0.93	0.77	1.12	.460
	Weighted median	12	-0.10	0.90	0.79	1.03	.140
	**IVW**	**12**	**-0.12**	**0.89**	**0.80**	**0.97**	**.013**
ebi-a-GCST90002023	MR Egger	7	0.07	1.07	0.58	1.95	.838
	Weighted median	7	0.35	1.42	1.08	1.87	.013
	**IVW**	**7**	**0.38**	**1.47**	**1.20**	**1.80**	**.000**
ebi-a-GCST90002057	MR Egger	12	-0.23	0.80	0.56	1.13	.235
	Weighted median	12	-0.20	0.82	0.69	0.96	.014
	**IVW**	**12**	**-0.24**	**0.78**	**0.64**	**0.96**	**.019**
ebi-a-GCST90002062	MR Egger	14	-0.20	0.82	0.58	1.14	.259
	Weighted median	14	-0.19	0.83	0.67	1.02	.077
	**IVW**	**14**	**-0.27**	**0.76**	**0.63**	**0.93**	**.008**
ebi-a-GCST90002091	MR Egger	9	0.22	1.24	1.03	1.51	.062
	Weighted median	9	0.14	1.15	1.02	1.29	.017
	**IVW**	**9**	**0.12**	**1.13**	**1.03**	**1.25**	**.014**

The bold values highlight the results of the primary analysis method employed in our study, namely the inverse-variance weighted (IVW) method. This is the standard and often most reliable approach for the main analysis in Mendelian randomization studies.

beta = the estimated causal effect of the exposure factor on the outcome, Exposure = ID sequences of the immune cells, IVW = inverse-variance weighted, nsnp = the number of instrumental variable SNPs, or = odds ratio, or_lci95 = the lower bound of the 95% confidence interval for the odds ratio, or_uci95 = the upper bound of the 95% confidence interval for the odds ratio, pval = *P*-value.

### 3.3. Causal effects between gut microbiome exposure and immune cell outcomes

We performed two-sample MR analysis between 14 gut microbiome types and 28 immune cell types, which were selected as described above. After completing 392 two-sample MR tests, the results from the IVW method indicated causal effects between 17 combinations of gut microbiome types and immune cells. All results passed pleiotropy and heterogeneity tests, as shown in Table S7, Supplemental Digital Content, https://links.lww.com/MD/P582.

### 3.4. Mediation analysis results

**Figure 2.**
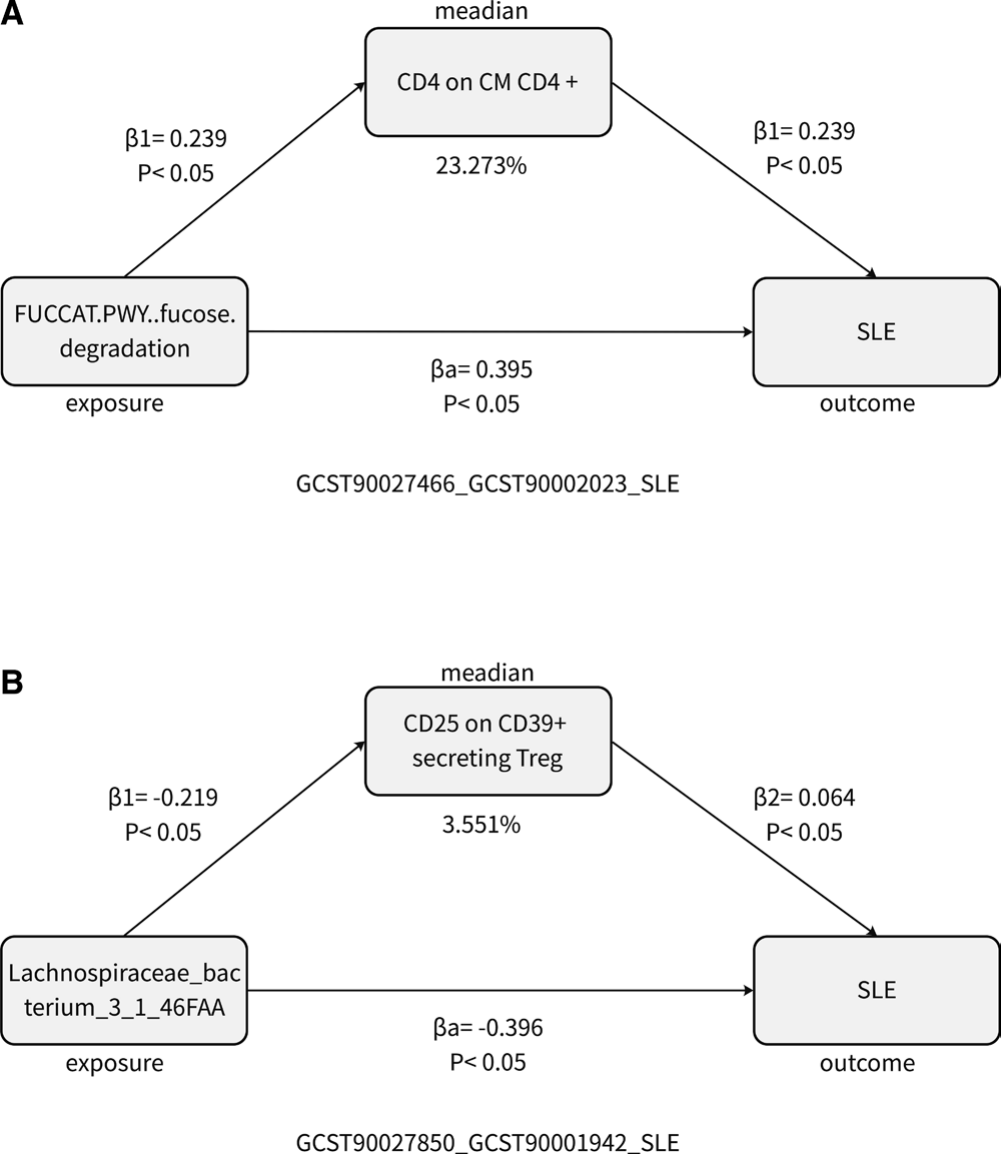
Mediation analysis results. (A) CD4 on CM CD4+ mediates 23.273% of the causal effect of FUCCAT.PWY.fucose.degradation on SLE. β1 = βGCST90027466_GCST900 02023, β2 = βGCST9000 2023_SLE, βa = βGCST90027466_SLE. Mediation proportion = β1 × β2/βa = 23.273%. (B) CD25 on CD39+ secreting Treg mediates 3.551% of the causal effect of Lachnospiraceae_bacterium_3_1_46FAA on SLE. β1 = βGCST90027850_GCST90001942, β2 = βGCST90001942_SLE, βa = βGCST 90027850_SLE. Mediation proportion = β1 × β2/βa = 3.551%. SLE = systemic lupus erythematosus.

**Figure 3.**
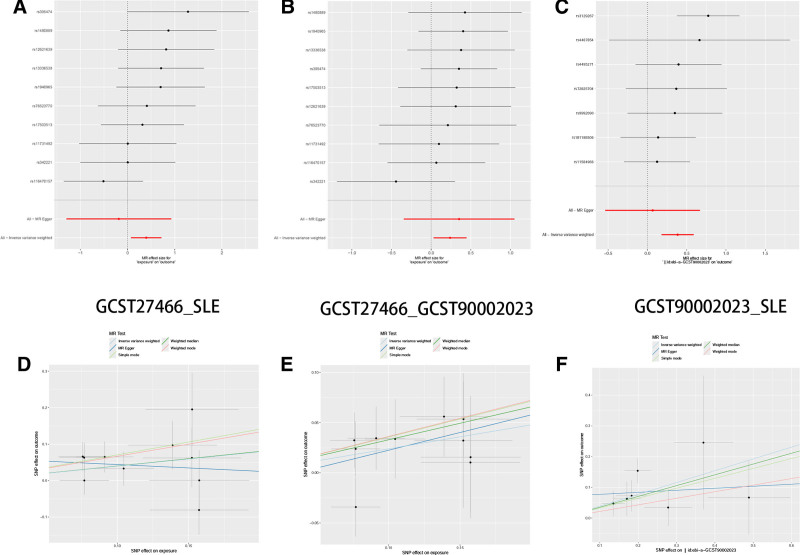
Visualization of Mendelian randomization analysis results. (A) Forest plot of SNPs for GCST27466_SLE. (B) Forest plot of SNPs for GCST27466_GCST90002023. (C) Forest plot of SNPs for GCST90002023_SLE. (D) Scatter plot of different MR analysis results for GCST27466_SLE. (E) Scatter plot of different MR analysis results for GCST27466_GCST90002023. (F) Scatter plot of different MR analysis results for GCST90002023_SLE. SLE = systemic lupus erythematosus.

**Figure 4.**
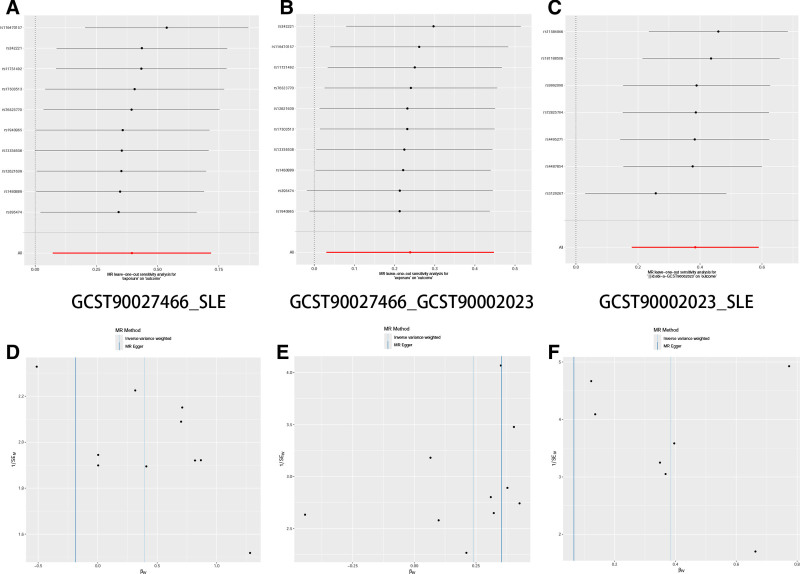
Visualization of sensitivity analysis results. (A) Funnel plot of GCST27466_SLE. (B) Funnel plot of GCST27466_GCST90002023. (C) Funnel plot of GCST90002023_SLE. (D) Scatter plot of GCST27466_SLE. (E) Scatter plot of GCST27466_GCST90002023. (F) Scatter plot of GCST90002023_SLE. SLE = systemic lupus erythematosus.

**Figure 5.**
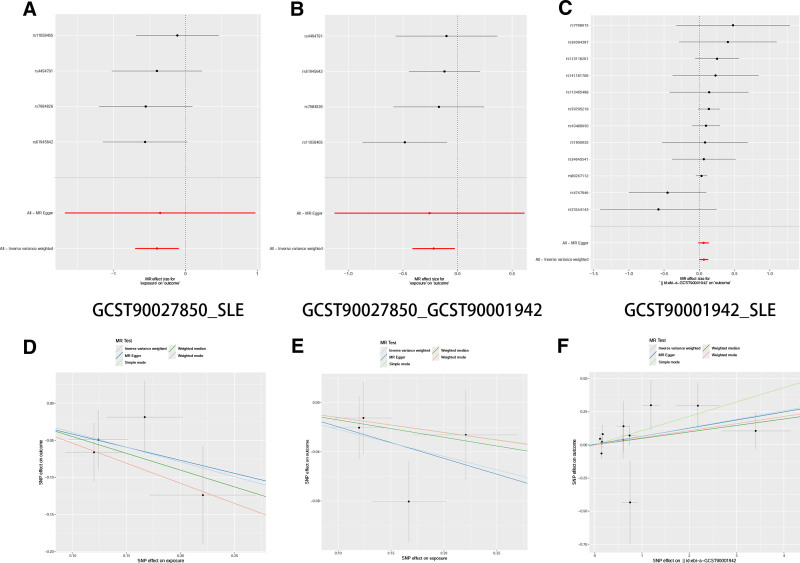
Visualization of Mendelian randomization analysis results. (A) Forest plot of SNPs for GCST27850_SLE. (B) Forest plot of SNPs for GCST27850_GCST90001942. (C) Forest plot of SNPs for GCST90001942_SLE. (D) Scatter plot of different MR analysis results for GCST27850_SLE. (E) Scatter plot of different MR analysis results for GCST27850_GCST90001942. (F) Scatter plot of different MR analysis results for GCST90001942_SLE. SLE = systemic lupus erythematosus.

**Figure 6.**
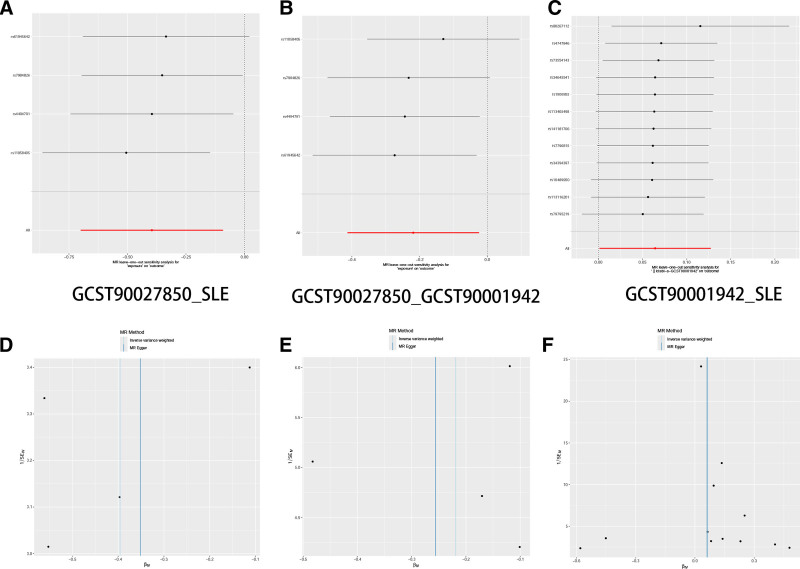
Visualization of sensitivity analysis results. (A) Funnel plot of GCST27850_SLE. (B) Funnel plot of GCST27850_GCST90 0019 42. (C) Funnel plot of GCST90001942_SLE. (D) Scatter plot of GCST27850_SLE. (E) Scatter plot of GCST27850_GCST90001942. (F) Scatter plot ofGCST90001942_SLE. SLE = systemic lupus erythematosus.

Before performing the mediation analysis on the 17 combinations of gut microbiome types and immune cells, we first used sign calculation to filter out combinations where the mediation effect sign was inconsistent with the total effect, eliminating 15 combinations with confounding effects. Subsequently, we conducted mediation analysis on the remaining 2 combinations of gut microbiome types and immune cells (GCST90027466_GCST90002023, GCST90027850_GCST90001942), and the results are presented in Table 3 (the IDs corresponding to each gut microbiota and immune cells are detailed in Tables S8 and S9, Supplemental Digital Content, https://links.lww.com/MD/P582). The analysis revealed that the GCST90027466 gut microbiome type (FUCCAT.PWY.fucose.degradation, β = 0.395 [0.072, 0.72], *P* = .016) increased the expression of the GCST90002023 immune cell type (CD4 on CM CD4+) and thereby enhanced the risk of SLE. Notably, CD4 on CM CD4+ mediated 23.273% of the causal effect of FUCCAT.PWY.fucose.degradation on SLE (Fig. 2A). Exposure to the GCST90027850 microbiota specie (Lachnospiraceae_bacterium_3_1_46FAA, β = ‐0.396 [‐0.700, ‐0.091], *P* = .011) reduces the expression of immune cell GCST90001942 type (CD25 on CD39+ secreting Treg), thereby lowering the risk of SLE. CD25 on CD39+ secreting Treg mediates 3.551% of the causal effect of Lachnospiraceae_bacterium_3_1_46FAA on SLE (Fig. 2B). The SNP instrument variable selection results for the 2 causal pathways are shown in Figs. 3A–C and 4A–C. The results and directions of different MR analyses are presented in Figs. 3D–F and 4D–F. Sensitivity analysis results are shown in Figs. 5A–C and 6A–C, and heterogeneity analysis results are shown in Figs. 5D–F and 6D–F.

**Table 3:** Two combinations of gut microbiome types exposure and immune cell mediators after selection.

Exposure (Gut microbiota)_meadian (immunocyte)	Method	nsnp	beta	or	or_lci95	or_uci95	pval
**GCST90027466_GCST90002023**	MR Egger	10	0.35	1.42	0.71	2.86	.351
	Weighted- median	10	0.33	1.39	1.06	1.82	.017
	**IVW**	**10**	**0.24**	**1.27**	**1.03**	**1.56**	**.025**
**GCST90027850_GCST90001942**	MR Egger	4	‐0.26	0.77	0.32	1.85	.622
	Weighted- median	4	‐0.14	0.87	0.67	1.12	.277
	**IVW**	**4**	‐**0.22**	**0.80**	**0.66**	**0.98**	**.027**

The bold values highlight the results of the primary analysis method employed in our study, namely the inverse-variance weighted (IVW) method. This is the standard and often most reliable approach for the main analysis in Mendelian randomization studies.

beta = the estimated causal effect of the exposure factor on the outcome, IVW = inverse-variance weighted, nsnp = the number of instrumental variable SNPs, or = odds ratio, or_lci95 = the lower bound of the 95% confidence interval for the odds ratio, or_uci95 = the upper bound of the 95% confidence interval for the odds ratio, pval = *P*-value.

## 4. Discussion

Systemic lupus erythematosus is a multifactorial disease with an unknown etiology, and its pathogenic mechanisms are extremely complex. Existing studies have shown that various factors, including the gut microbiome, contribute to the onset and progression of the disease.^[13]^ Through metabolic products and the complex communication mechanisms between the host’s gastrointestinal system and microbiota, the gut microbiota engages in “spatiotemporal dialogs” with the host, regulating and influencing a series of physiological and pathological processes. This interaction plays an important role in the pathogenesis of autoimmune diseases such as SLE, where immune cells serve as key mediators in regulating the occurrence and development of autoimmune diseases. Notably, similar gut–immune axis dysregulation has been observed in other immune-mediated disorders like immune thrombocytopenia, where gut microbiota-derived exosomes activate complement pathways to promote platelet destruction, highlighting conserved mechanisms across diseases.^[14]^ Our study found that CD4+ cells on CM CD4+ and CD25+ on CD39+ secreting Tregs mediate the causal effects of the FUCCAT.PWY.fucose.degradation and Lachnospiraceae_ bacterium_ 3_1_46FAA gut microbiome types in SLE.

### 4.1. Exposure to the FUCCAT.PWY.fucose.degradation increases the expression of CD4 on CM CD4+ cells, thereby elevating the risk of systemic lupus erythematosus

Our research found that the exposure to FUCCAT.PWY.fucose.degradation increases the risk of SLE, which is consistent with previous studies.^[5]^ FUCCAT.PWY is the core pathway for the microbial degradation of L-fucose, converting this rare sugar into energy (ATP) and metabolic precursors (such as DHAP) through a series of metabolic processes. The specific role of fucose in gut homeostasis and autoimmune diseases remains controversial. Some studies suggest that fucose plays a significant positive role in maintaining gut homeostasis. These studies propose that fucose protects intestinal immune balance by promoting the colonization of beneficial gut bacteria and reducing the toxic metabolic products of specific microbiota. In the context of autoimmune enteropathy, it has been found that fucose can alter the metabolic pathways of certain pathogens, such as *Fusobacterium nucleatum*, in the gut, thereby suppressing their pro-inflammatory characteristics.^[15]^ However, other researches indicates that fucose also plays an important role in the pathogenic effects of harmful bacteria on the gut. Fucose can act as a mediator for the infection of harmful bacteria such as *Helicobacter pylori*, and can promote the colonization and toxicity of pathogenic bacteria like *Escherichia coli* by modulating specific metabolic pathways.^[16,17]^ This may be attributed to the widespread distribution of fucose metabolism pathways. Studies have shown that various harmful and beneficial microbiota, including *Salmonella enterica* serovar Typhimurium and *Clostridium difficile*, can utilize fucose as a carbon source.^[18]^ Therefore, further research is needed to delineate the specific roles of different subgroups of microbiota in disease.

Our study also reveals the causal effect of the FUCCAT.PWY.fucose.degradation mediated by CD4 on CM CD4 in SLE, a finding not previously reported in the literature. “CD4 on CM CD4” refers to central memory T cells (CM T cells), which are a subset of memory T cells generated from naive T cells after antigen activation. These cells possess long-term memory and can home to lymph nodes to receive further antigen stimulation.^[19]^ In patients with SLE, memory T cells undergo significant expansion and, through various mechanisms such as the release of pro-inflammatory cytokines and the promotion of the activation and differentiation of autoreactive T cells, contribute to immune imbalance and the development of autoimmunity.^[20,21]^ Additionally, memory T cells can infiltrate damaged tissues, leading to chronic organ and tissue damage.^[22,23]^ In multiple affected organs, such as the skin and kidneys, infiltration of memory T cells has been observed in SLE patients.^[24]^ However, memory T cells, including CM T cells, encompass several subtypes at different stages of development, and there is currently a lack of research exploring the similarities and differences in the pathogenic mechanisms of these different memory T cell subtypes. Our study suggests that exposure to CD4 on CM CD4 immunecell may exacerbate the risk of SLE, and further research in this area could uncover new therapeutic targets for SLE treatment.

### 4.2. Exposure to Lachnospiraceae bacterium 3_1_46FAA reduces the expression of CD25 on CD39+ secreting Treg cells, thereby decreasing the risk of systemic lupus erythematosus

Our research found that exposure to the microbiota specie Lachnospiraceae bacterium 3_1_46FAA reduces the risk of SLE, which is consistent with previous studie.^[19]^ Lachnospiraceae is considered a potentially beneficial bacterium, producing short-chain fatty acids, such as butyrate, which have significant anti-inflammatory properties and play an important role in maintaining intestinal and immune health.^[25,26]^ Previous studies have indicated that a reduction in Lachnospiraceae in the gut is associated with the development of immune-related diseases, such as chronic spontaneous urticaria and SLE.^[27]^ Sequencing studies of the microbiota in the intestines of SLE patients have found that reduced platelet counts in SLE patients are linked to a decrease in the abundance of Lachnospiraceae.^[28]^ This suggests that Lachnospiraceae plays a protective role in the pathogenesis of SLE; however, related studies have not yet explored the specific mechanisms behind its protective effect.

Our research found that Lachnospiraceae bacterium 3_1_46FAA reduce the expression of CD25+ CD39+ secretory Treg cells, thereby lowering the risk of SLE. This mechanism has not been reported in previous studies. Treg cells are crucial for maintaining immune homeostasis and peripheral tolerance. Traditional studies have suggested that Treg cells primarily play a protective role in autoimmune diseases such as SLE.^[29]^ However, recent researches indicates that the role of Treg cells in SLE may be controversial, as Treg cells of different subtypes exhibit varying alterations in SLE.^[30]^ Some studies show that Treg cells have significant adaptability in different tissue microenvironments and can develop effector functions similar to those of conventional T cells under specific conditions.^[30]^ Altered Treg cells, which secrete pro-inflammatory cytokines and display effector characteristics similar to those of helper T cells, have been detected in patients with autoimmune diseases such as multiple sclerosis, inflammatory bowel disease, SLE, and rheumatoid arthritis.^[31]^ This may reflect the complexity and heterogeneity of the roles of different Treg subpopulations in the mechanisms of these diseases. Therefore, further studies exploring the similarities and differences in the roles of various Treg subtypes, including CD25+ CD39+ secretory Treg cells, in the pathogenesis of SLE are essential.

In summary, our study demonstrates the causal effect of immune cells mediating the casual effect of gut microbiome on SLE. As the first investigation into the relationship between gut microbiome, immune cells, and SLE, this study provides new insights and theoretical foundations for the development of therapeutic targets for the disease. However, there are some limitations of our study. Due to the lack of clinical biological samples, we were unable to conduct more in-depth mechanistic research to clarify the specific regulatory mechanisms and intervention strategies involved in this process. We hope that future studies will integrate genomic, transcriptomic, metabolomic, and proteomic data to comprehensively unravel the complex relationships between gut microbiome, immune cells, and SLE, particularly addressing the contradictions present in existing researches. Moreover, further investigation into how environmental factors (such as diet and lifestyle) and genetic factors (such as HLA gene polymorphisms) interact with gut microbiome and immune cells will help deepen our understanding of the disease’s heterogeneous mechanisms, ultimately identifying new targets for diagnosis and treatment.

## 5. Conclusion

Through large-scale MR and mediation analysis, we identified the causal effect of immune cells mediating the gut microbiome on SLE. Furthermore, 2 specific causal effect pathways were selected, which further validate the impact of the gut microbiome on host immune balance. These findings provide a theoretical foundation for the development of diagnostic and therapeutic strategies for the disease.

## Acknowledgments

The authors would like to thank Lopera-Maya, Orrù V, and Kurki MI for uploading their meaningful an valuable datasets.

## Author contributions

**Conceptualization:** Bo Zhao.

**Formal analysis:** Jia Zhang.

**Funding acquisition:** Hongye Wang, Jiyu Chen, Bo Zhao.

**Investigation:** Xiran Yang.

**Software:** Mifeng Yang.

**Validation:** Na Yang, Bo Zhao.

**Visualization:** Jiyu Chen.

**Writing – original draft:** Hongye Wang.

**Writing – review & editing:** Jia Zhang.

## Supplementary Material


